# Supplementary material for: Validation of Three MicroScan® Antimicrobial Susceptibility Testing Plates Designed for Low-Resource Settings
Source: Diagnostics (Basel). 2022 Aug 30;12(9):2106. doi: 10.3390/diagnostics12092106 (PMC9497938; doi:10.3390/diagnostics12092106)
Supplement: Supplementary file 1 [file diagnostics-12-02106-s001.zip › diagnostics-1852496-supplementary.pdf]

Table S1. List of antibiotics or resistance mechanisms tests included onto the different MSF MIC panels as pers WHO AWaRe category and based on the list of essential medicine

			MIC POS Panel[1]		MIC NEG Panel[2]		MIC FAST Panel[3]	
Antibiotic	Class	AWaRe Category	*Staphylo-cocci*	*Entero-cocci*	*Enterobac-terales*	*Non-Fer-menting bacilli*	*Strepto-cocci*	*Haemoph-ilus*
Ampicillin	Penicillins	Access	X	X	X		X	
Benzylpenicillin	Penicillins	Access	X				X	
Oxacillin	Penicillins	Access	X					
Amoxicillin/clavulanic Acid	Beta lactam - beta lactamase inhibitor	Access			X			
Chloramphenicol	Amphenicols	Access			X		X	X
Gentamicin	Aminoglycosides	Access	X		X	X		
Gentamicin (high level)	Aminoglycosides	Access		X				
Amikacin	Aminoglycosides	Access	X		X	X		
Clindamycin	Lincosamides	Access	X					
Inducible clindamycin resistance	Lincosamides	Access	X					
Tetracycline	Tetracyclines	Access	X					
Trimethoprim/sulfamethoxazole	Trimethoprim	Access	X		X	X	X	X
Cefoxitin screen	Second-generation cephalosporins	Watch	X					
Ceftazidime	Third generation cephalosporins	Watch			X	X		
Ceftriaxone	Third generation cephalosporins	Watch			X		X	X
ESBL test	Third generation cephalosporins	Watch			X	X		
Piperacillin/tazobactam	Beta lactam - beta lactamase inhibitor	Watch			X	X		
Ertapenem	Carbapenems	Watch			X	X		
Imipenem/cilastatin	Carbapenems	Watch			X	X		
Meropenem	Carbapenems	Watch			X	X	X	X
Ciprofloxacin	Fluoroquinolones	Watch	X	X	X	X		X
Levofloxacin	Fluoroquinolones	Watch					X	X
Teicoplanin	Glycopeptides	Watch	X	X				
Vancomycin (IV)	Glycopeptides	Watch	X	X			X	
Erythromycin	Macrolides	Watch	X					
Tigecycline	Glycylcyclines	Reserve	X	X				
Daptomycin	Lipopeptides	Reserve	X					
Linezolid	Oxazolidinones	Reserve	X				X	
Fosfomycin (IV)	Phosphonics	Reserve	X		X			
Colistin	Polymyxins	Reserve			X	X		
Dalfopristin-quinupristin	Streptogramins	Reserve	X	X				

[1] MicroScan Inducible Clindamycin test is intended to detect inducible resistance for staphylococci with the antimicrobial agent clindamycin. The MicroScan Cefoxitin Screen is intended to determine the susceptibility of Staphylococcus species to the penicillinase-stable beta-lactams. The Cefoxitin Screen uses the 16-20 hour result from a well containing cefoxitin at 4 mcg/mL and growth media, labeled CfxS, and the oxacillin MIC at 16-20 hours..

[2] Trimethoprim, and Trimethoprim/Sulfamethoxazole broth contain thymidine phosphorylase to reduce thymidine levels in the medium. Ceftazidime, cefotaxime, ceftazidime/ clavulanic acid and cefotaxime/clavulanic acid are used to confirm the presence of ESBLs. The confirmation test is a ≥ 3 twofold dilution decrease in MICs of suspected organisms to ceftazidime or cefotaxime in the presence of a fixed concentration of clavulanic acid, versus its MIC when tested alone.

[3] Panels are rehydrated with Mueller-Hinton broth supplemented with 5% lysed horse blood (LHB) for Streptococci as recommended by EUCAST and with Hemophilus Test Medium (HTM) for Hemophilus testing as recommended by CLSI, after inoculation of the broth with a standardized suspension of the organism.

Table S2.a) MicroScan MSF Dried Overnight Gram-Negative panel are designed for use in determining antimicrobial agent susceptibility of aerobic and facultatively anaerobic gram-negative bacilli (Enterobacteriacea and non-fermenting Gram negative bacilli)

C	G			8 AMP	16 AMP	32 AMP	64 AMP	2/2 AMC	8/2 AMC	16/2 AMC	32/2 AMC
8/4 PIT	16/4 PIT	32/4 PIT	64/4 PIT	2 CTA	16 CTA	1 CTZ	4 CTZ	8 CTZ	16 CTZ		
				0.5/4 CTXAC	4/4 CTXAC	0.25/4 CCV	2/4 CCV	1 CTR	2 CTR	4 CTR	8 CTR
0.06 CIP	0.12 CIP	0.25 CIP	0.5 CIP	1 CIP	2 CIP						
8 AMI	16 AMI	32 AMI	2 GEN	4 GEN	8 GEN	16 GEN	1 TIG	2 TIG	4 TIG	8 TIG	
4 CHL	8 CHL	16 CHL	2 COL	4 COL	32 FOS	64 FOS	2/38 TRS	4/76 TRS	8/152 TRS		
0.12 MER	0.25 MER	0.5 MER	1 MER	2 MER	4 MER	8 MER	16 MER				
0.5 IMI	1 IMI	2 IMI	4 IMI	8 IMI	16 IMI	0.12 ERT	0.25 ERT	0.5 ERT	1 ERT	2 ERT	4 ERT

Abbr.	Antimicrobial Agents	Dilutions
C	Negative Control	
G	Growth Control	
AMI	Amikacin	8-32
AMC	Amoxicillin/Clavulanic Acid	2/2, 8/2 - 32/2
AMP	Ampicillin	8 - 64
CTA	Cefotaxime	2, 16
CTXAC	Cefotaxime/Clavulanic Acid	0.5/7, 4/4
CTZ	Ceftazidime	1, 4 -16
CCV	Ceftazidime/Clavulanic	0.25/4, 2/4
CTR	Ceftriaxone	1 - 8
CHL	Chloramphenicol	4 - 16
CIP	Ciprofloxacin	0.06 - 2
COL	Colistin	2 - 4
ERT	Ertapenem	0.12 - 4
FOS	Fosfomycin	32 - 64
GEN	Gentamicin	2 - 16
IMI	Imipenem	0.5 - 16
MER	Meropenem	0.12 - 16
PIT	Piperacillin/Tazobactam	8/4 - 64/4
TIG	Tigecycline	1 - 8
TRS	Trimethoprim/Sulfamethoxazole	2 / 38 - 8/152

Table S2.b) MicroScan MSF Dried Overnight Gram Positive panel are designed for the determination of antimicrobial agent susceptibility of rapidly growing aerobic and facultative gram-positive cocci (Staphylococcus.spp and Enterococcus.spp)

C	G		TFG	0.12<br>PEN	0.25<br>PEN	0.5<br>PEN	1<br>PEN	4<br>AMP	8<br>AMP	16<br>AMP	32<br>AMP
0.25<br>OXA	0.5<br>OXA	1<br>OXA	2<br>OXA	4<br>CfxS	0.5<br>CIP	1<br>CIP	2<br>CIP	4<br>CIP			
8<br>AMI	16<br>AMI	32<br>AMI	64<br>AMI	1<br>GEN	2<br>GEN	500<br>GmS					
2<br>TEI	4<br>TEI	8<br>TEI	16<br>TEI	2<br>VAN	4<br>VAN	8<br>VAN	16<br>VAN				
0.25<br>CLI	0.5<br>CLI	1<br>CLI	2<br>CLI	4/0.5<br>ICd	0.5<br>ERY	1<br>ERY	2<br>ERY	4<br>ERY	1<br>QUD	2<br>QUD	4<br>QUD
1<br>LIN	2<br>LIN	4<br>LIN	8<br>LIN								
1<br>DAP	2<br>DAP	4<br>DAP	8<br>DAP	32<br>FOS	64<br>FOS	0.5/9.5<br>TRS	1/19<br>TRS	2/38<br>TRS	4/76<br>TRS		
1<br>TET	2<br>TET	4<br>TET	8<br>TET	0.25<br>TIG	0.5<br>TIG	1<br>TIG	2<br>TIG				

Abbr.	Antimicrobial Agents	Dilutions
C	Negative Control	
G	Growth Control	
TFG	Thymidine Free Growth Well	(Comparator for SXT)
AMI	Amikacin	8 - 64
AMP	Ampicillin	4 - 32
CfxS	Cefoxitin Screen	4
CIP	Ciprofloxacin	0.5 - 4
CLI	Clindamycin	0.25 - 2
DAP	Daptomycin	1 - 8
ERY	Erythromycin	0.5 - 4
FOS	Fosfomycin	32 - 64
GEN	Gentamicin	1 - 2
GmS	Gentamicin Synergy Screen	500
Icd	Inducible Clindamycin Test	4 / 0.5
LIN	Linezolid	1 - 8
OXA	Oxacillin	0.25 - 2
PEN	Penicillin	0.12 - 1
QUD	Quinupristin/Dalfopristin	1 - 4
TEI	Teicoplanin	2 - 15
TET	Tetracycline	1 - 8
TIG	Tigecycline	0.25 - 2
TRS	Trimethoprim/Sulfamethoxazole	0.5 / 9.5 - 4 / 76
VAN	Vancomycin	2 - 16

Table S2.c) MicroScan MSF Dried Overnight Fastidious panel are designed for use in determining antimicrobial agent susceptibility of aerobic non-enterococcal streptococci (including Streptococcus pneumoniae) and Haemophilus spp. Not to be used for testing of Neisseria

G											
0.06 PEN	0.12 PEN	0.25 PEN	0.5 PEN	1 PEN	2 PEN						
0.12 AMP	0.5 AMP	1 AMP	2 AMP	0.12 CTR	0.5 CTR	1 CTR	2 CTR	0.25 MER	0.5 MER	1 MER	2 MER
0.06 CIP	0.12 CIP	0.5 LEV	2 LEV	4 LEV							
2 VAN	4 VAN	8 VAN	16 VAN								
0.5 CLI	1 CLI	2 CLI	4 CLI								
2 LIN	4 LIN	8 LIN	16 LIN								
2 CHL	4 CHL	8 CHL	16 CHL	0.5/9.5 TRS	1/19 TRS	2/38 TRS	4/76 TRS				

Abbr.	Antimicrobial Agents	Dilutions
C	Negative Control	
NOTE = No Negative Control Well (C) because broth is added to the entire panel when inoculated		
AMP	Ampicillin – AMP	0.12 , 0.5 - 2
CTR	Ceftriaxone – CTR	0.12 , 0.5 - 2
CHL	Chloramphenicol – CHL	2 - 16
CIP	Ciprofloxacin – CIP	0.06 - 0.12
CLI	Clindamycin – CLI	0.5 - 4
LEV	Levofloxacin – LEV	0.5, 2 - 4
LIN	Linezolid – LIN	2 - 16
MER	Meropenem – MER	0.25 - 2
PEN	Penicillin – PEN	0.06 - 2
CIP	Trimethoprim/Sulfamethoxazole – TRS	0.5/9.5 - 4/76
VAN	Vancomycin – VAN	2 - 16

Table S3. Technical information on the different panel, disc, media used for the experiment (Lot number, reference, expiry date, etc.)

Item	Method	Cond	Brand	Ref	Batch	Date exp
Pos MIC Panel Type 1	BMD	20/box	BC	B1020-103B	19190329	2020-01-10
Neg MIC MSF Panel Type 1	BMD	20/box	BC	B1020-104B	19190330	2020-01-10
FAST MSF RUO MIC Panel Type 1	BMD	20/box	BC	B1020-105W	19190331	2020-02-10
Prompt	Inoculum	60/box	BC	B1026-10D	19170714	2020-01-10
Cation-adjusted Mueller-Hinton Broth with 3% Lysed Horse Blood, 25mL (BC)	Broth	10/box	BC	B1015-25	19190328	2020-01-10
Haemophilus Test Medium (HTM), 25mL	Broth	10/box	BC	B1015-26	19190326	2020-03-10
Inoculator-D Set, 60/pk (BC)	Consumable	260/box	BC	B1013-5	19190324	2023-04-10
Inoculum Water, 3mL (BC)	Inoculum	60/box	BC	B1015-2	19190907	2020-04-10
Inoculum Water with Pluronic, 25 mL	Inoculum	60/box	BC	B1015-7	19190928	2020-04-10
Amikacin	30µg	4 x 50 Disc	Biorad	66148	64240285	2019-12-10
Amoxicillin/Clavulanic Acid	20-10µg	4 x 50 Disc	Biorad	66178	200417A	2019-10
Ampicillin	2µg	4 x 50 Disc	Biorad	67288	64239659	2019-11-27
Ampicilline	10µg	4 x 50 Disc	Biorad	66128	64239660	2019-11-12
Cefepime	30µg	4 x 50 Disc	Biorad	66098	64261585	2020-03-17
Cefepime/Clavulanic Acid	30µg/10µg	4 x 50 Disc	Biorad	66466	190117G	2020-01
Cefotaxime	5µg	4 x 50 Disc	Biorad	67718	64253166	2020-02-11
Cefotaxime/Clavulanic Acid	5µg/10µg	4 x 50 Disc	Biorad	68456	190117N	2020-01
Cefoxitin Screen	30 µg	4 x 50 Disc	Biorad	66228	200318A	2020-04
Ceftazidime	10µg	4 x 50 Disc	Biorad	67298	64251000	2020-01-27
Ceftazidime/Clavulanic Acid	30µg/10µg	4 x 50 Disc	Biorad	68446	64251001	2020-01-27
Ceftriaxone	30µg	4 x 50 Disc	Biorad	66188	200414C	2020-04
Chloramphenicol	30 µg	4 x 50 Disc	Biorad	66278	64221108	2020-09-11
Ciprofloxacin	5µg	4 x 50 Disc	Biorad	68648	64240327	2020-02-17
Clindamycin	2 µg	4 x 50 Disc	Biorad	66328	64246826	2020-01-03
Colistin	BMD	8*10	Fisher	FRCOL	B1441A	2020-01
Ertapenem	10µg	4 x 50 Disc	Biorad	67518	64248425	2019-02-14
Erythromycin	15 µg	4 x 50 Disc	Biorad	66448	64231553	2019-11-05
AD Fosfomycin 0.25-256	Agar dilution	6 test	Liofilchem	77061	22122083	2020-12-18
Gentamicin	10µg	4 x 50 Disc	Biorad	66608	64246826	2020-03-18
Gentamicin Synergy Screen	30µg	4 x 50 Disc	Biorad	667318	64182638	2020-03-25
Imipenem	10µg	4 x 50 Disc	Biorad	66568	4331644	2020-10-31
Levofloxacin	5µg	4 x 50 Disc	Biorad	66858	4331644	2020-10-31
Linezolid	10 µg	4 x 50 Disc	Biorad	67878	200417D	2020-07
Meropenem	10µg	4 x 50 Disc	Biorad	67048	64261586	2020-03-17
Penicillin	1 unit	4 x 50 Disc	Biorad	67788	64226720	2019-10-08
Piperacillin/Tazobactam	30-6µg	4 x 50 Disc	Biorad	67338	64248373	2020-01-13
Quinupristin/Dalfopristin	15 µg	4 x 50 Disc	Biorad	67528	64221134	2020-10-03
Teico/ Vanco / Dapto MIC	BMD	10	Fisher	FRUNIGP1	B1341B	2020-08
Teicoplanin	30µg	4 x 50 Disc	Biorad	68948	64240292	2019-12-12
Tetracycline	30µg	4 x 50 Disc	Biorad	67448	64240328	2020-01-01
Tigecycline	15µg	4 x 50 Disc	Biorad	67398	64248382	2020-02-10
Trimethoprim/Sulfa	1.25-23.75µg	4 x 50 Disc	Biorad	68898	64240288	2019-12-19
Vancomycin	5µg	4 x 50 Disc	Biorad	68888	64219266	2020-08-28
Mueller-Hinton-F square	Agar	10 plaques	Biorad	63525	64468399	2020-02
Mueller-Hinton square	Agar	10 plaques	Biorad	63901	64460701	2020-05
Chocolate agar w/o Vitox	Agar	20 plates 90 mm	Liofilchem	10601	22122082	2019-10

Table S4: Results for Staphylococci tested with MICPOS1 using Prompt inoculum method and visual reading versus AST reference panels

		No. of isolates *AST Reference*[b]			No. of isolates MICPOS1[c]						
Antimicrobial	Total[a]	R	I	S	R	I	S	PtCA[d] (no. [%])	PtmE[e] (no. [%])	PtME[f] (no. [%])	PtVME[g] (no. [%])
Penicillin	63	59	0	4	59	0	4	63(100)	0 (0)	0 (0)	0 (0)
Ciprofloxacin	74	37	0	37	43	0	31	68 (92)	0 (0)	6 (8)	0 (0)
Amikacin	74	17	4	53	18	4	52	73 (99)	0 (0)	1 (1)	0 (0)
Gentamicin	74	28	0	46	33	11	30	58 (78)	11 (15)	5 (7)	0 (0)
Teicoplanin	74	3	0	71	10	0	64	67 (91)	0 (0)	7 (9)	0 (0)
Vancomycin	74	2	0	72	4	0	70	72 (97)	0 (0)	2 (3)	0 (0)
Quinupristin-dalfopristin	74	0	7	67	2	7	65	70 (95)	0 (0)	3 (4)	1 (1)
Erythromycin	74	30	0	44	30	0	44	74 (100)	0 (0)	0 (0)	0 (0)
Clindamycin	74	14	0	60	20	1	53	65 (88)	1 (1)	7 (9)	1 (1)
Daptomycin	74	1	0	73	1	0	73	72 (97)	0 (0)	1 (1)	1 (1)
Fosfomycin	74	29	0	45	29	0	45	74 (100)	0 (0)	0(0)	0 (0)
Trimethoprim/Sulfamethoxazole	74	18	0	56	29	0	55	71 (96)	0 (0)	2 (3)	1 (1)
Linezolid	74	5	0	69	6	0	68	73 (99)	0 (0)	1 (1)	0 (0)
Tetracycline	74	37	0	37	47	4	23	60 (81)	4 (5)	10 (14)	0 (0)
Tigecycline	74	0	0	74	4	0	70	70 (95)	0 (0)	4 (5)	0 (0)

a Total number of isolates tested by antibiotic
b Number of isolate tested classified with reference method by R resistant; I, intermediate; S, susceptible.
c Number of isolate tested classified with evaluated method by R resistant; I, intermediate; S, susceptible
d PtCA, categorical agreement with PROMPT inoculum.
e PtmE, minor error with PROMPT inoculum.
f PtME, major error with PROMPT inoculum.
g PtVME, very major error with PROMPT inoculum.

Table S5: Results for Enterococci tested with MICPOS1 using Prompt inoculum method and visual reading versus AST reference panels

		No. of isolates *AST Reference*[b]			No. of isolates MICPOS1[c]						
Antimicrobial	Total[a]	R	I	S	R	I	S	PtCA[d] (no. [%])	PtmE[e] (no. [%])	PtME[f] (no. [%])	PtVME[g] (no. [%])
Ampicillin	49	30	0	19	29	1	18	47 (96)	0 (0)	1 (2)	1 (2)
Ciprofloxacin	49	29	0	20	30	0	19	48 (98)	0 (0)	1 (2)	0 (0)
Teicoplanin	49	23	0	26	23	0	26	47 (96)	0(0)	1 (2)	1 (2)
Vancomycin	49	23	0	26	23	0	26	49 (100)	0 (0)	0 (0)	0 (0)
Quinupristin-dalfopristin	49	19	16	14	19	16	14	49 (100)	0 (0)	0(0)	0 (0)
Linezolid	49	2	0	47	2	7	40	42 (91)	7 (9)	0 (0)	0 (0)
Tigecycline	49	4	0	45	4	0	45	49 (100)	0 (0)	0 (0)	0 (0)

a Total number of isolates tested by antibiotic
b Number of isolate tested classified with reference method by R resistant; I, intermediate; S, susceptible.
c Number of isolate tested classified with evaluated method by R resistant; I, intermediate; S, susceptible
d PtCA, categorical agreement with PROMPT inoculum.
e PtmE, minor error with PROMPT inoculum.
f PtME, major error with PROMPT inoculum.
g PtVME, very major error with PROMPT inoculum.

Table S6: Results for Enterobacterales tested with MICNEG1 using Prompt inoculum method and visual reading versus AST reference panels

Antimicrobial	Total[a]	No. of isolates *AST Reference*[b] R	I	S	No. of isolates MICPNEG1[c] R	I	S	PtCA[d] (no. [%])	PtmE[e] (no. [%])	PtME[f] (no. [%])	PtVME[g] (no. [%])
Ampicillin	112	100	0	12	99	0	13	111 (99)	0 (0)	0 (0)	1 (1)
Amoxicillin/Clavulanic Acid	112	72	0	40	73	0	39	109 (97)	0 (0)	2 (2)	1 (1)
Ceftazidime	112	47	2	63	49	0	64	108 (96)	3 (3)	1 (1)	0 (0)
Ceftriaxone	112	50	0	62	51	1	60	110 (98)	1 (1)	1 (1)	0 (0)
Piperacillin/Tazobactam	112	39	4	69	39	4	69	112 (100)	0 (0)	0 (0)	0 (0)
Ciprofloxacin	112	51	4	57	54	8	49	107 (95)	3 (3)	3 (3)	0 (0)
Amikacin	112	16	4	92	18	11	83	104 (92)	7 (6)	2 (2)	0 (0)
Gentamicin	112	40	5	67	40	8	64	108 (96)	3 (3)	1 (1)	1 (1)
Trimethoprim/Sulfamethoxazole	112	69	1	42	70	2	40	106 (94)	1 (1)	3 (3)	2 (2)
Chloramphenicol	112	47	0	65	46	1	65	108 (96)	1 (1)	1 (1)	2 (2)
Colistin	112	12	0	77	15	0	74	109 (97)	0 (0)	3 (3)	0 (0)
Fosfomycin	112	1	0	111	2	0	110	109 (97)	0 (0)	2 (2)	1 (1)
Tigecycline	112	0	0	112	0	0	112	112 (100)	0 (0)	0 (0)	0 (0)
Meropenem	112	16	6	90	16	6	90	112 (100)	0 (0)	0 (0)	0 (0)
Imipenem	112	19	7	86	20	12	90	107 (95)	5 (4)	1 (1)	0 (0)
Ertapenem	112	33	0	79	34	6	84	106 (94)	6 (5)	1 (1)	0 (0)

a Total number of isolates tested by antibiotic
b Number of isolate tested classified with reference method by R resistant; I, intermediate; S, susceptible.
c Number of isolate tested classified with evaluated method by R resistant; I, intermediate; S, susceptible
d PtCA, categorical agreement with PROMPT inoculum.
e PtmE, minor error with PROMPT inoculum.
f PtME, major error with PROMPT inoculum.
g PtVME, very major error with PROMPT inoculum.

Table S7: Results for Non-fermenting Gram negative bacilli tested with MICNEG1 using Prompt inoculum method and visual reading versus AST reference panels

Antimicrobial	Total[a]	No. of isolates *AST Reference*[b] R	I	S	No. of isolates MICPNEG1[c] R	I	S	PtCA[d] (no. [%])	PtmE[e] (no. [%])	PtME[f] (no. [%])	PtVME[g] (no. [%])
Ceftazidime	15	8	3	4	8	3	4	15 (100)	0 (0)	0 (0)	0 (0)
Piperacillin/Tazobactam	15	4	0	11	4	0	11	15 (100)	0 (0)	0 (0)	0 (0)
Ciprofloxacin	29	12	6	11	12	6	11	29 (100)	0 (0)	0 (0)	0 (0)
Amikacin	29	7	2	20	7	3	19	28 (97)	1 (3)	0 (0)	0 (0)
Gentamicin	29	15	0	14	15	0	14	29 (100)	0 (0)	0 (0)	0 (0)
Trimethoprim/Sulfamethoxazole	29	10	0	20	10	0	20	30 (100)	0 (0)	0 (0)	0 (0)
Chloramphenicol	9	4	3	2	4	3	2	9 (100)	0 (0)	0 (0)	0 (0)
Colistin	15	3	0	12	3	0	12	15 (100)	0 (0)	0 (0)	0 (0)
Meropenem	45	14	6	25	14	8	23	45 (96)	2 (4)	0 (0)	0 (0)

Imipenem	29	12	0	17	12	0	17	29 (97)	0 (0)	0 (0)	1 (3)

a Total number of isolates tested by antibiotic
b Number of isolate tested classified with reference method by R resistant; I, intermediate; S, susceptible.
c Number of isolate tested classified with evaluated method by R resistant; I, intermediate; S, susceptible
d PtCA, categorical agreement with PROMPT inoculum.
e PtmE, minor error with PROMPT inoculum.
f PtME, major error with PROMPT inoculum.
g PtVME, very major error with PROMPT inoculum.

Table S8: Results for specific resistance test using MICNEG1 or MICPOS1 using Prompt inoculum method and visual reading versus AST reference methods

Multidrug resistant organism	No. of isolates *AST Reference*[b]			No. of isolates MSF panel[c]		PtCA[d] (no. [%])	PtME[e] (no. [%])	PtVME[f] (no. [%])
	Total[a]	R	S	R	S			
Methycillin resistant *Staphylococcus aureus*[g]	47	33	14	34	13	46 (99)	1 (1)	0 (0)
Inducible clindamycin resistant Staphyloccocci[g]	74	26	48	26	48	74 (100)	0 (0)	0(0)
Vancomycin resistant *Staphylococcus aureus*[g]	47	2	45	4	43	43 (96)	3 (4)	1 (1)
High-level gentamicin resistance Enterococci[g]	49	25	24	25	24	47 (100)	1 (2)	1 (2)
Vancomycin resistant Enterococci[g]	49	23	26	23	26	49 (100)	0 (0)	0(0)
Extented spectrum beta lactamase Enterobacterales[h]	112	19	93	19	93	112 (100)	0 (0)	0(0)
Carbapenem resistant Enterobacterales[h]	112	33	79	32	80	0 (0)	0 (0)	1 (1)
Colistin resistant Enterobacterales[h]	112	12	77	13	76	112 (97)	2 (2)	1 (1)
Carbapenem resistant *Pseudomonas aeruginosa*[h]	15	4	11	4	11	15 (100)	0 (0)	0 (0)
Colistin resistant *Pseudomonas aeruginosa*[h]	15	4	11	4	11	15 (100)	0 (0)	0 (0)
Carbapenem resistant *Acinetobacter baumannii*[h]	14	8	6	7	7	15 (100)	0 (0)	1 (3)

a Total number of isolates tested by antibiotic
b Number of isolate tested classified with reference method by R resistant; I, intermediate; S, susceptible.
c Number of isolate tested classified with evaluated method by R resistant; I, intermediate; S, susceptible
d PtCA, categorical agreement with PROMPT inoculum.
e PtME, major error with PROMPT inoculum.
f PtVME, very major error with PROMPT inoculum.
g Resistance test evaluated on the MICPOS1 panel.
h Resistance test evaluated on the MICNEG1 panel.

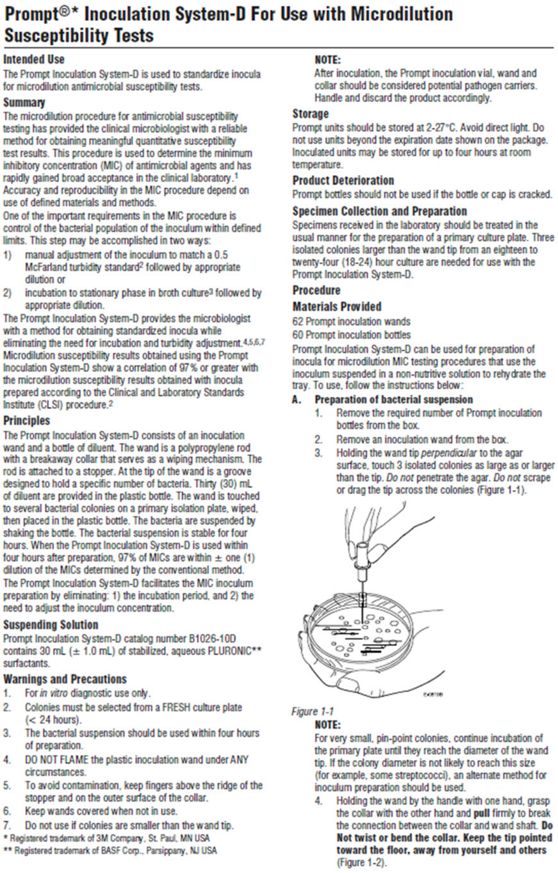

Prompt®* Inoculation System-D For Use with Microdilution Susceptibility Tests

Intended Use

The Prompt Inoculation System-D is used to standardize inocula for microdilution antimicrobial susceptibility tests.

Summary

The microdilution procedure for antimicrobial susceptibility testing has provided the clinical microbiologist with a reliable method for obtaining meaningful quantitative susceptibility test results. This procedure is used to determine the minimum inhibitory concentration (MIC) of antimicrobial agents and has rapidly gained broad acceptance in the clinical laboratory.[1] Accuracy and reproducibility in the MIC procedure depend on use of defined materials and methods.

One of the important requirements in the MIC procedure is control of the bacterial population of the inoculum within defined limits. This step may be accomplished in two ways:

1) manual adjustment of the inoculum to match a 0.5 McFarland turbidity standard[2] followed by appropriate dilution or
2) incubation to stationary phase in broth culture[3] followed by appropriate dilution.

The Prompt Inoculation System-D provides the microbiologist with a method for obtaining standardized inocula while eliminating the need for incubation and turbidity adjustment.[4,5,6,7] Microdilution susceptibility results obtained using the Prompt Inoculation System-D show a correlation of 97% or greater with the microdilution susceptibility results obtained with inocula prepared according to the Clinical and Laboratory Standards Institute (CLSI) procedure.[2]

Principles

The Prompt Inoculation System-D consists of an inoculation wand and a bottle of diluent. The wand is a polypropylene rod with a breakaway collar that serves as a wiping mechanism. The rod is attached to a stopper. At the tip of the wand is a groove designed to hold a specific number of bacteria. Thirty (30) mL of diluent are provided in the plastic bottle. The wand is touched to several bacterial colonies on a primary isolation plate, wiped, then placed in the plastic bottle. The bacteria are suspended by shaking the bottle. The bacterial suspension is stable for four hours. When the Prompt Inoculation System-D is used within four hours after preparation, 97% of MICs are within ± one (1) dilution of the MICs determined by the conventional method. The Prompt Inoculation System-D facilitates the MIC inoculum preparation by eliminating: 1) the incubation period, and 2) the need to adjust the inoculum concentration.

Suspending Solution

Prompt Inoculation System-D catalog number B1026-10D contains 30 mL (± 1.0 mL) of stabilized, aqueous PLURONIC** surfactants.

Warnings and Precautions

1. For *in vitro* diagnostic use only.
2. Colonies must be selected from a FRESH culture plate (< 24 hours).
3. The bacterial suspension should be used within four hours of preparation.
4. DO NOT FLAME the plastic inoculation wand under ANY circumstances.
5. To avoid contamination, keep fingers above the ridge of the stopper and on the outer surface of the collar.
6. Keep wands covered when not in use.
7. Do not use if colonies are smaller than the wand tip.

* Registered trademark of 3M Company, St. Paul, MN USA
** Registered trademark of BASF Corp., Parsippany, NJ USA

NOTE:
After inoculation, the Prompt inoculation vial, wand and collar should be considered potential pathogen carriers. Handle and discard the product accordingly.

Storage

Prompt units should be stored at 2-27°C. Avoid direct light. Do not use units beyond the expiration date shown on the package. Inoculated units may be stored for up to four hours at room temperature.

Product Deterioration

Prompt bottles should not be used if the bottle or cap is cracked.

Specimen Collection and Preparation

Specimens received in the laboratory should be treated in the usual manner for the preparation of a primary culture plate. Three isolated colonies larger than the wand tip from an eighteen to twenty-four (18-24) hour culture are needed for use with the Prompt Inoculation System-D.

Procedure

Materials Provided

62 Prompt inoculation wands
60 Prompt inoculation bottles

Prompt Inoculation System-D can be used for preparation of inocula for microdilution MIC testing procedures that use the inoculum suspended in a non-nutritive solution to rehydrate the tray. To use, follow the instructions below:

A. Preparation of bacterial suspension

1. Remove the required number of Prompt inoculation bottles from the box.
2. Remove an inoculation wand from the box.
3. Holding the wand tip *perpendicular* to the agar surface, touch 3 isolated colonies as large as or larger than the tip. *Do not* penetrate the agar. *Do not* scrape or drag the tip across the colonies (Figure 1-1).

Figure 1-1

NOTE:
For very small, pin-point colonies, continue incubation of the primary plate until they reach the diameter of the wand tip. If the colony diameter is not likely to reach this size (for example, some streptococci), an alternate method for inoculum preparation should be used.

4. Holding the wand by the handle with one hand, grasp the collar with the other hand and **pull** firmly to break the connection between the collar and wand shaft. **Do Not twist or bend the collar. Keep the tip pointed toward the floor, away from yourself and others** (Figure 1-2).

(a)

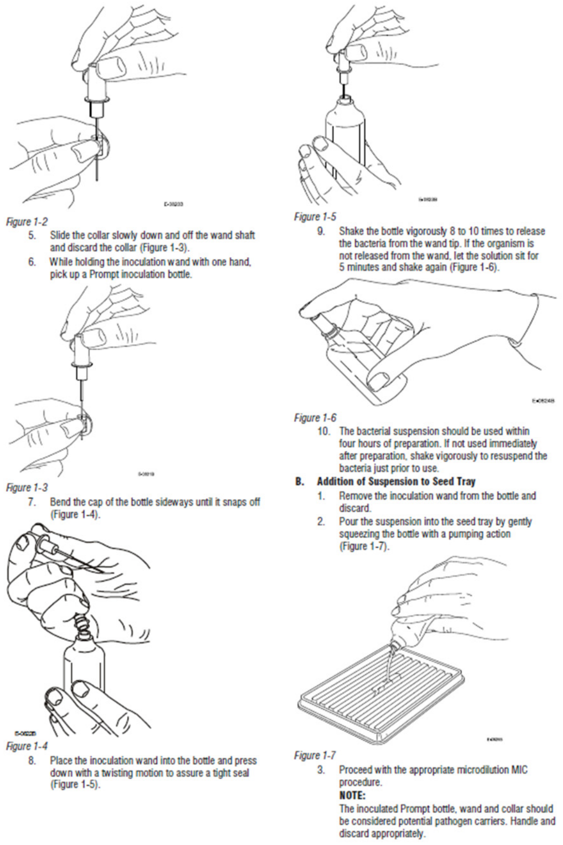

Figure 1-2

5. Slide the collar slowly down and off the wand shaft and discard the collar (Figure 1-3).
6. While holding the inoculation wand with one hand, pick up a Prompt inoculation bottle.

Figure 1-3

7. Bend the cap of the bottle sideways until it snaps off (Figure 1-4).

Figure 1-4

8. Place the inoculation wand into the bottle and press down with a twisting motion to assure a tight seal (Figure 1-5).

Figure 1-5

9. Shake the bottle vigorously 8 to 10 times to release the bacteria from the wand tip. If the organism is not released from the wand, let the solution sit for 5 minutes and shake again (Figure 1-6).

Figure 1-6

10. The bacterial suspension should be used within four hours of preparation. If not used immediately after preparation, shake vigorously to resuspend the bacteria just prior to use.

B. Addition of Suspension to Seed Tray

1. Remove the inoculation wand from the bottle and discard.
2. Pour the suspension into the seed tray by gently squeezing the bottle with a pumping action (Figure 1-7).

Figure 1-7

3. Proceed with the appropriate microdilution MIC procedure.
   NOTE:
   The inoculated Prompt bottle, wand and collar should be considered potential pathogen carriers. Handle and discard appropriately.

(b)

Figure S1. Extraction of the Prompt® Inoculation System-D Instruction For Use with Microdilution Susceptibility Tests with, (a) general instructions and (b) bench aid like part with more comprehensive instructions

Table S9: Results of the repetition done for AMC discrepant results using different AST methods and brand for disc, media. Were used in this experiment disc from Liofilchem(Liofilchem, Roseto degli Abruzzi, Italy), Biorad, I2A; MH agar from Biorad, Liofilchem, Biomerieux MH Enterobacterales, E-test Gradiant strip (E-test, bioMérieux, Marcy l'Ètoile, France) and for Broth Microdilution Sensititre (Sensititre, Thermo Fisher Scientific, UK), MICNEG1 (Microscan, Beckman Coulter

N° isolat	Type of method Brand Disc / Brand agar	BMD MIC-NEG1		BMD Sensi-titre		GD E test/ Bio		KB Lio/Bio		KB I2A/Bio		KB Bio/Bio		KB Lio/Lio		KB I2A/Lio		KB Bio/Lio		KB Lio/BM		KB I2A/BM		KB Bio/BM	
128	*Salmonella typhimurium*	16	R	>8	R	4	S	21	S	21	S	21	S	21	S	19	S	21	S	18	R	17	R	19	S
129	*Salmonella typhimurium*	16	R	>8	R	6	S	20	S	17	S	20	S	20	S	18	R	21	S	19	S	16	R	19	S
130	*Salmonella cholerasuis*	16	R	>8	R	8	S	21	S	20	S	21	S	21	S	18	R	21	S	18	R	17	R	20	S
131	*Salmonella cholerasuis*	16	R	>8	R	3	S	20	S	18	R	21	S	22	S	18	R	22	S	20	S	17	R	23	S
133	*Salmonella cholerasuis*	16	R	>8	R	3	S	23	S	19	S	24	S	23	S	20	S	24	S	21	S	17	R	22	S
135	*Salmonella cholerasuis*	8	R	>8	R	3	S	20	S	19	S	22	S	22	S	21	R	22	S	15	R	22	S	23	S
138	*Salmonella typhimurium*	32	R	>8	R	4	S	18	R	17	R	19	R	18	R	17	S	19	S	19	S	15	R	19	S
139	*Salmonella typhimurium*	32	R	>8	R	6	S	17	R	15	R	17	R	17	R	15	S	18	R	16	R	14	R	17	R
140	*Salmonella typhimurium*	32	R	>8	R	4	S	19	S	17	R	20	S	21	S	17	S	21	S	19	S	17	R	19	S
141	*Salmonella typhimurium*	8	S	>8	R	4	S	20	S	18	R	21	S	21	S	18	S	20	S	19	S	19	S	20	S
142	*Salmonella typhimurium*	8	S	>8	R	4	S	22	S	20	S	22	S	21	S	19	R	23	S	21	S	18	R	21	S

*Abreviations: BMD= Broth Micro Dilution; GD= Gradient Diffusion; KB = Kirby Bauer; Bio = Biorad; Lio = Liofilchem; I2A=I2A

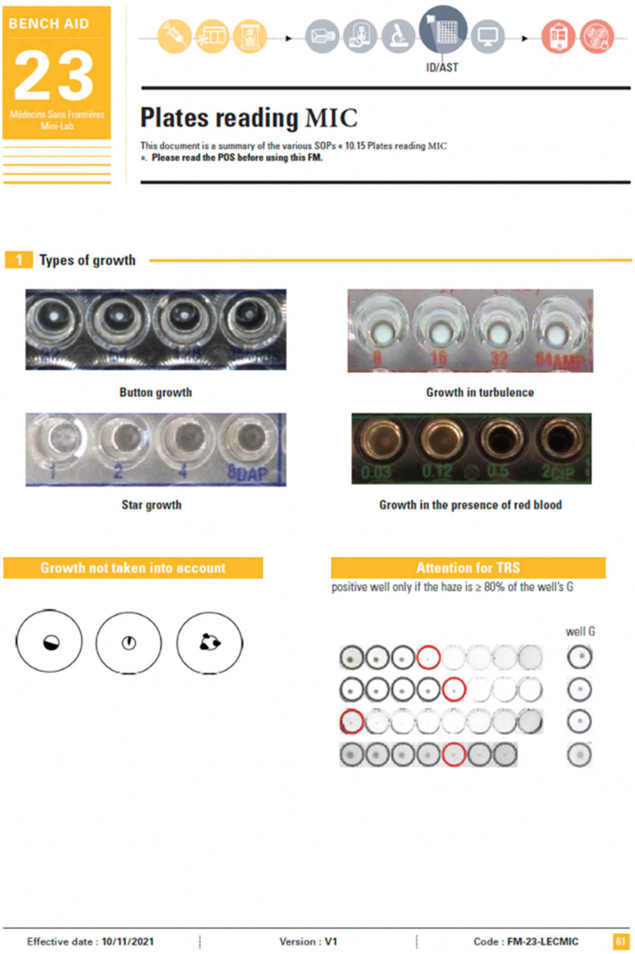

(a)

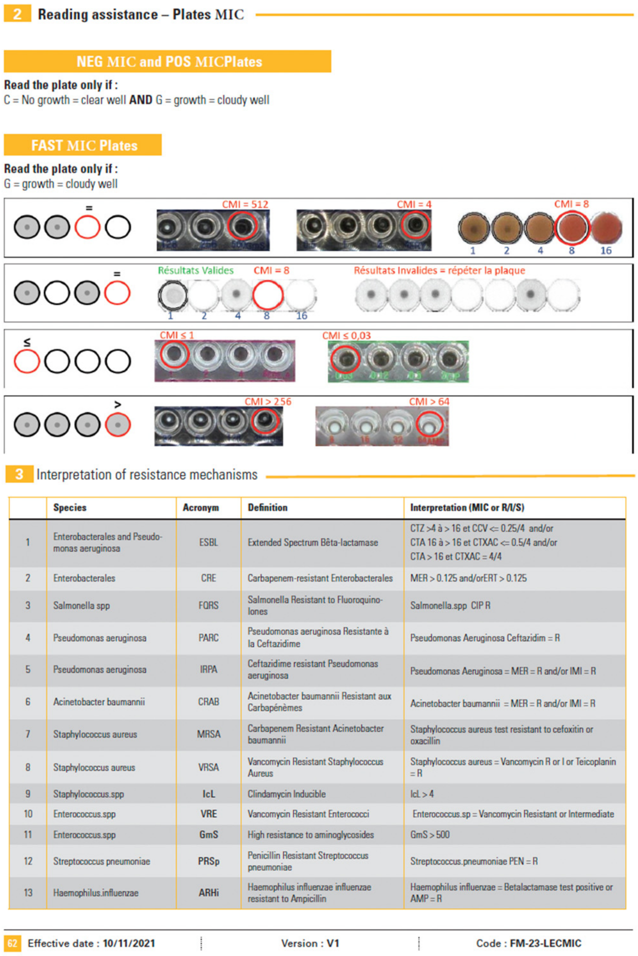

	Species	Acronym	Definition	Interpretation (MIC or R/I/S)
1	Enterobacterales and Pseudo-monas aeruginosa	ESBL	Extended Spectrum Bêta-lactamase	CTZ >4 à > 16 et CCV <= 0.25/4 and/or<br>CTA 16 à > 16 et CTXAC <= 0.5/4 and/or<br>CTA > 16 et CTXAC = 4/4
2	Enterobacterales	CRE	Carbapenem-resistant Enterobacterales	MER > 0.125 and/orERT > 0.125
3	Salmonella spp	FQRS	Salmonella Resistant to Fluoroquino-lones	Salmonella.spp CIP R
4	Pseudomonas aeruginosa	PARC	Pseudomonas aeruginosa Resistante à la Ceftazidime	Pseudomonas Aeruginosa Ceftazidim = R
5	Pseudomonas aeruginosa	IRPA	Ceftazidime resistant Pseudomonas aeruginosa	Pseudomonas Aeruginosa = MER = R and/or IMI = R
6	Acinetobacter baumannii	CRAB	Acinetobacter baumannii Resistant aux Carbapénèmes	Acinetobacter baumannii = MER = R and/or IMI = R
7	Staphylococcus aureus	MRSA	Carbapenem Resistant Acinetobacter baumannii	Staphylococcus aureus test resistant to cefoxitin or oxacillin
8	Staphylococcus aureus	VRSA	Vancomycin Resistant Staphylococcus Aureus	Staphylococcus aureus = Vancomycin R or I or Teicoplanin = R
9	Staphylococcus.spp	IcL	Clindamycin Inducible	IcL > 4
10	Enterococcus.spp	VRE	Vancomycin Resistant Enterococci	Enterococcus.sp = Vancomycin Resistant or Intermediate
11	Enterococcus.spp	GmS	High resistance to aminoglycosides	GmS > 500
12	Streptococcus pneumoniae	PRSp	Penicillin Resistant Streptococcus pneumoniae	Streptococcus.pneumoniae PEN = R
13	Haemophilus.influenzae	ARHi	Haemophilus influenzae influenzae resistant to Ampicillin	Haemophilus influenzae = Betalactamase test positive or AMP = R

(b)

Figure S2. Extraction of Mini-Lab Bench aid user manual Version1 2022 with, (a) description of type of growth and (b) visual description of the identification of MIC and mechanism of resistance
